# Supplementary material for: A multi-lock inhibitory mechanism for fine-tuning enzyme activities of the HECT family E3 ligases
Source: Nat Commun. 2019 Jul 18;10:3162. doi: 10.1038/s41467-019-11224-7 (PMC6639328; doi:10.1038/s41467-019-11224-7)
Supplement: Supplementary file 1 — Supplementary Information [file 41467_2019_11224_MOESM1_ESM.pdf]

Supplementary Information

A multi-lock inhibitory mechanism for fine-tuning enzyme activities of the HECT family E3 ligases

Z. Wang, Z. Liu et al.

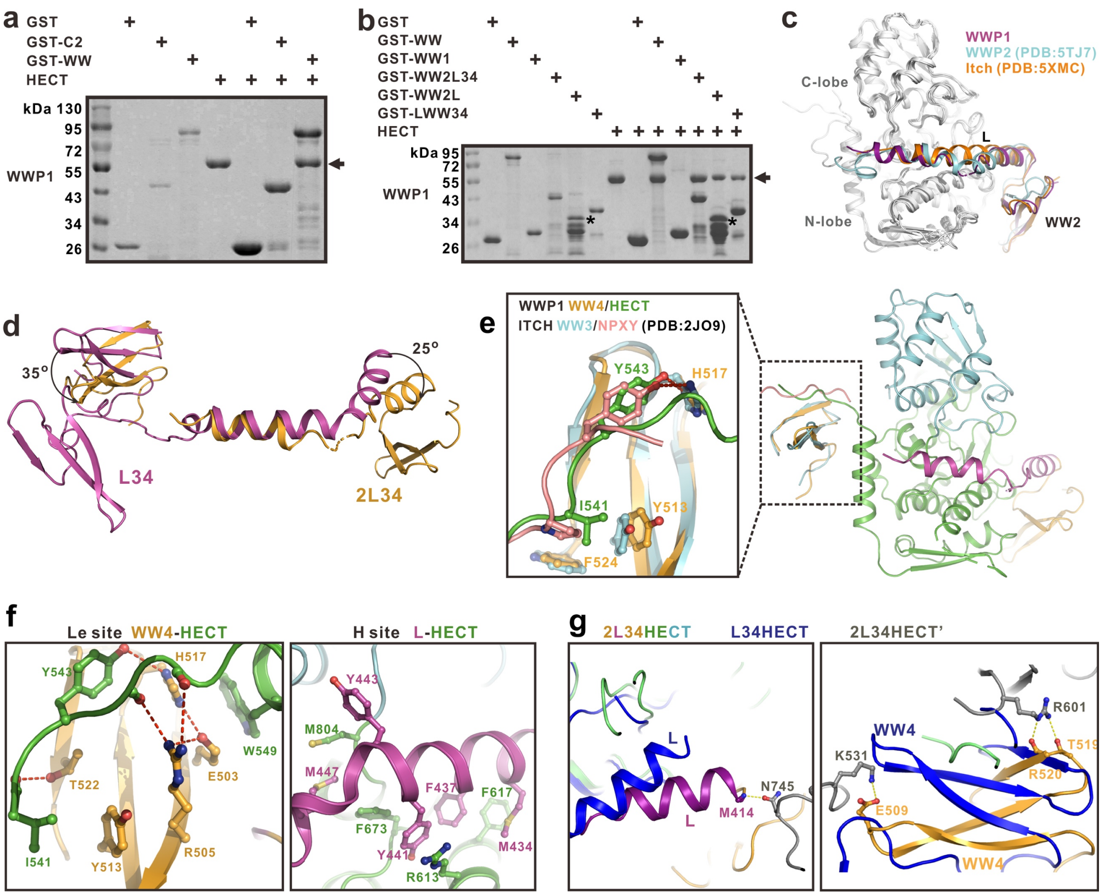

Supplementary Fig. 1 Autoinhibition mechanism of WWP1. (**a**) GST pull-down assay of GST-tagged WWP1 C2 or WW with Trx-HECT. (**b**) GST pull-down assay of GST-tagged WWP1 WW, WW1, WW2L34, WW2L or LWW34 with Trx-HECT. (**c**) Structural comparison of WWP1 2L34HECT (purple), WWP2 2LHECT (PDB ID: 5TJ7, cyan), and Itch 12L34HECT (PDB ID: 5XMC, orange). (**d**) Structural comparison of WWP1 2L34HECT (orange) and L34HECT (purple). For clarity, the HECT domains were removed from the structures. (**e**) Comparison of WW4-HECT and WW-PY motif interaction modes. The "IxY^{543}" motif from the N-terminal extension of WWP1 HECT perfectly occupied the canonical "NPxY" motif binding site of WW4. (**f**) The detailed structure of L-HECT and WW4-HECT interfaces from L34HECT. (**g**) Crystal contacts of 2L34HECT in the crystal. Left panel: M414 from L (magenta) of 2L34HECT interacts with N745 of a symmetric molecule (grey). Right panel: The main chains of T519 and R520 from WW4 (orange) of 2L34HECT form hydrogen bonds with R601 from a symmetric molecule (grey). E509 from WW4 (orange) forms a salt bridge with K531 from a symmetric molecule (grey). All these crystal contacts do not exist in the L34HECT (blue) structure. Source data are provided as a Source Data file.

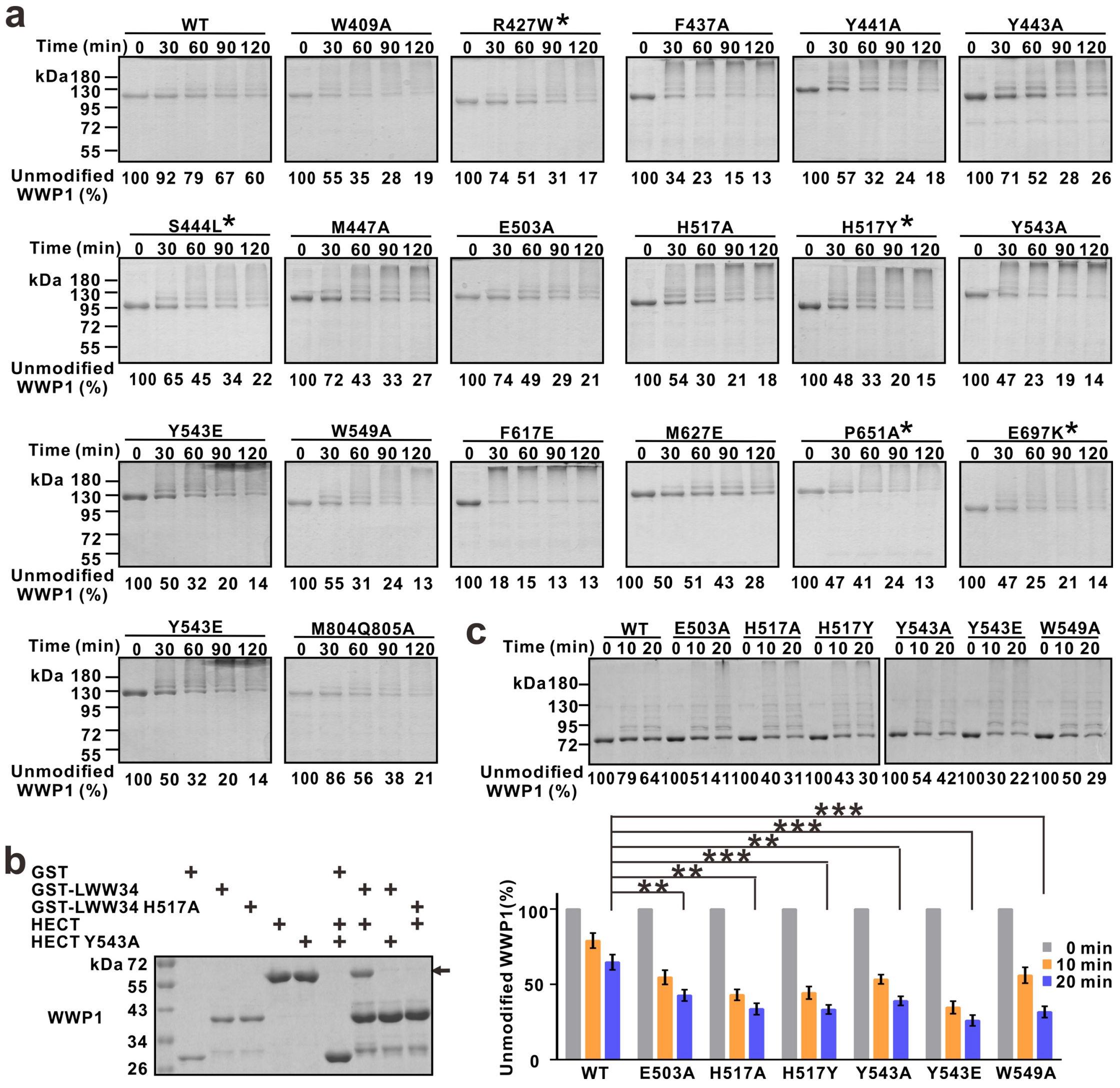

Supplementary Fig. 2 Autoubiquitination assay of WWP1 proteins. (**a**) Autoubiquitination assay of WWP1 12L34HCT WT and various mutants. Weight markers belong to all separate gels on the same line. Cancer-related mutants are marked with asterisk. (**b**) GST pull-down assay of WWP1 GST-LWW34 with Trx-HECT. Mutations that were predicted to impair interactions at the "Le" site in WW4-HECT ($Y543A^{HECT}$ and $H517A^{WW4}$) disrupted the interaction. (**c**) In vitro autoubiquitination assay of various WWP1 L34HECT mutants. Source data are provided as a Source Data file.

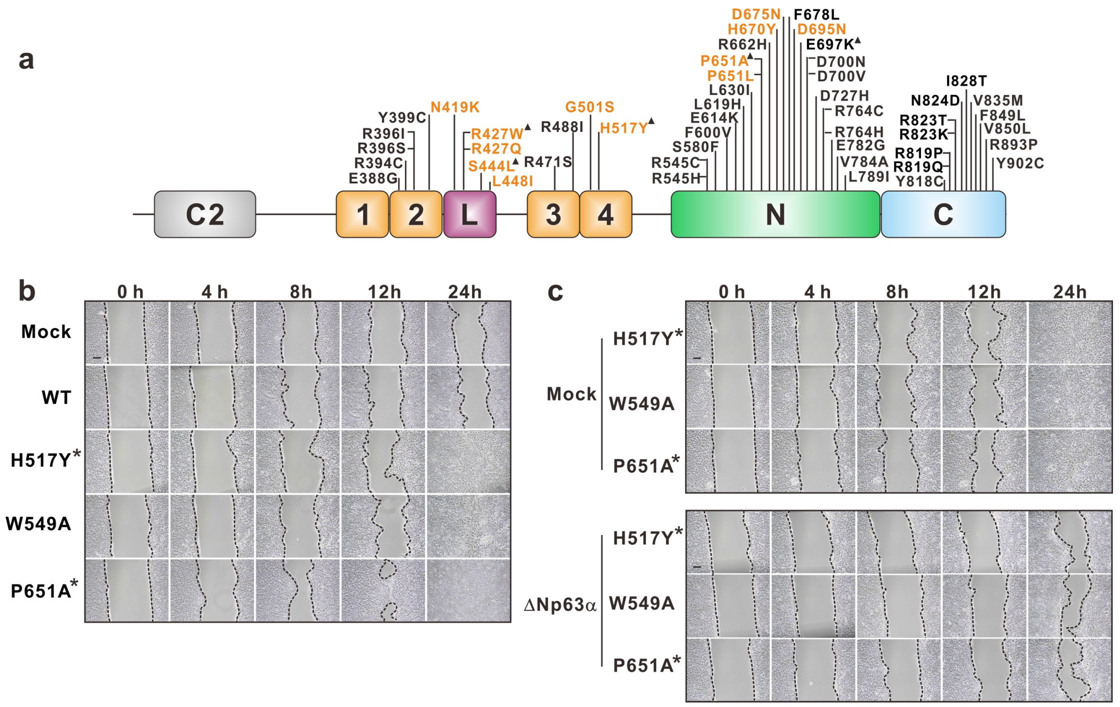

Supplementary Fig. 3 Cancer-related WWP1 mutations promote cell migration. (**a**) Schematic illustration for cancer-related mutations on WWP1 2L34HECT. Based on COSMIC cancer somatic mutation database, 85 out of 159 mutations of WWP1 fell into WW2L34HECT, among which 48 mutations could be explained by the WWP1 structure in this study and were labeled on the cartoon. Mutations located in the WW2L34-HECT interface were colored in yellow, and those critical for protein folding in black. Mutations validated by in vitro autoubiquitination assay and cell migration assay were highlighted with triangle. (**b**) Wound-healing assay of MCF-10A cells stably expressing WWP1 WT, various mutants or vector control (Mock). (**c**) ΔNp63α or vector control (Mock) were overexpressed in MCF-10A stable cells expressing various WWP1 mutants, and the cells were then subjected to wound-healing assay. Scale bars, 200 μm. Cancer-related mutants are marked with asterisk.

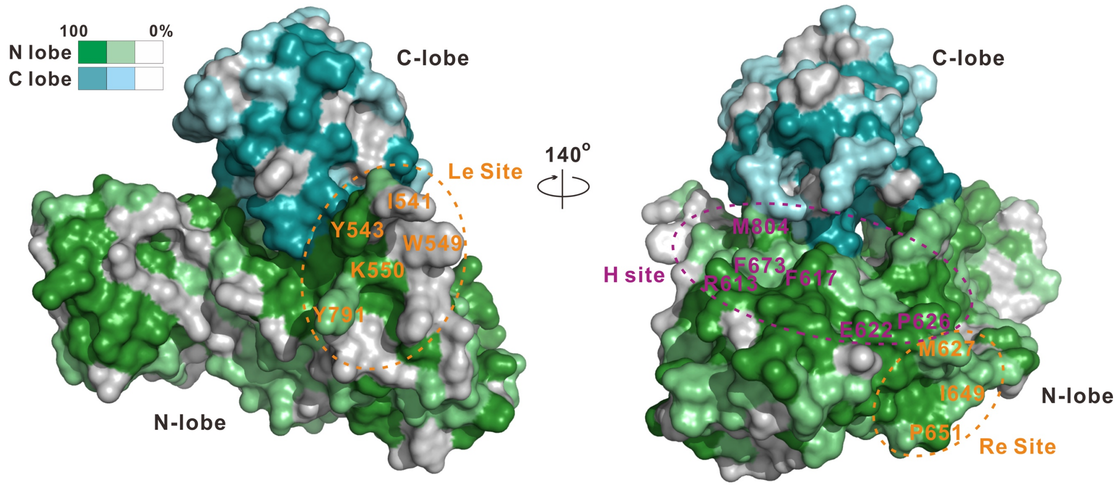

Supplementary Fig. 4 The conservation of the regulatory sites on HECT domain. Surface representations showing the amino acid sequence conservation map of HECT domains. The conservation map is calculated based on the sequence alignment of Nedd4 family E3s (human WWP1/2, Itch, Nedd4/4L, Smurf1/2, NEDL1/2, *Drosophila* Su(dx), Yeast Rsp5) and human HUWE1. Residues from the "Re", "Le", and "H" sites involved in autoinhibition of WWP1 were labeled on the map.

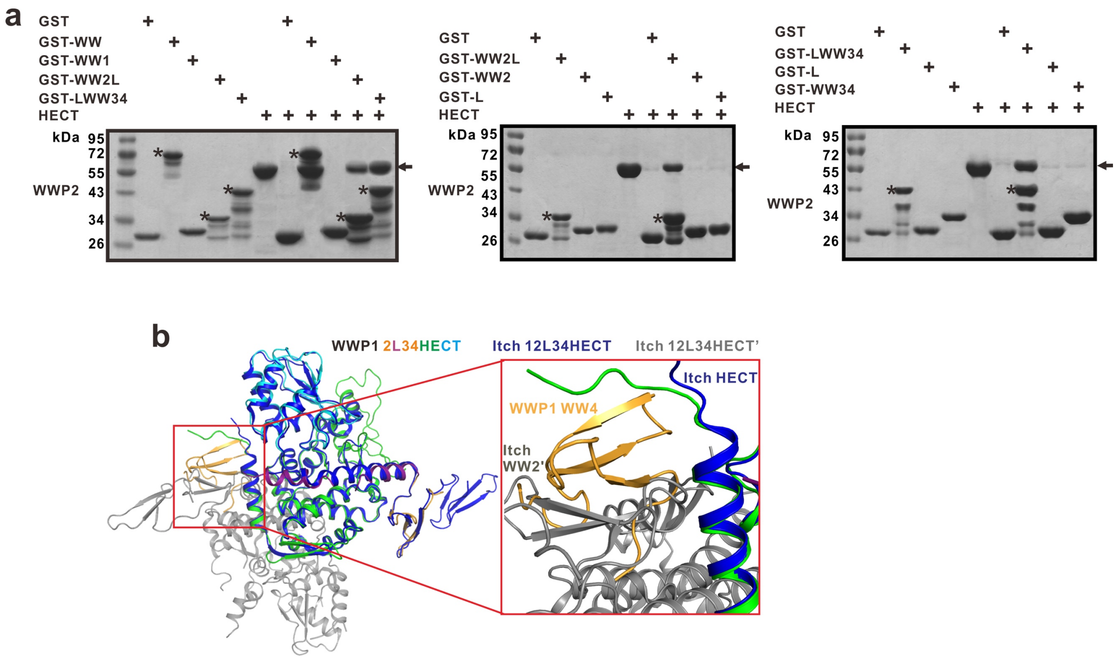

Supplementary Fig. 5 WWP2 and Itch might adopt the same multi-lock regulation mechanism as WWP1. (**a**) GST pull-down assay of GST-tagged WWP2 WW, WW1, WW2L, LWW34, WW2, L or WW34 with Trx-HECT. (**b**) Crystal contacts of Itch 12L34HECT (blue). The WW4 binding "Le" site in Itch is occupied by the WW2 domain of a symmetric molecule (grey). Source data are provided as a Source Data file.

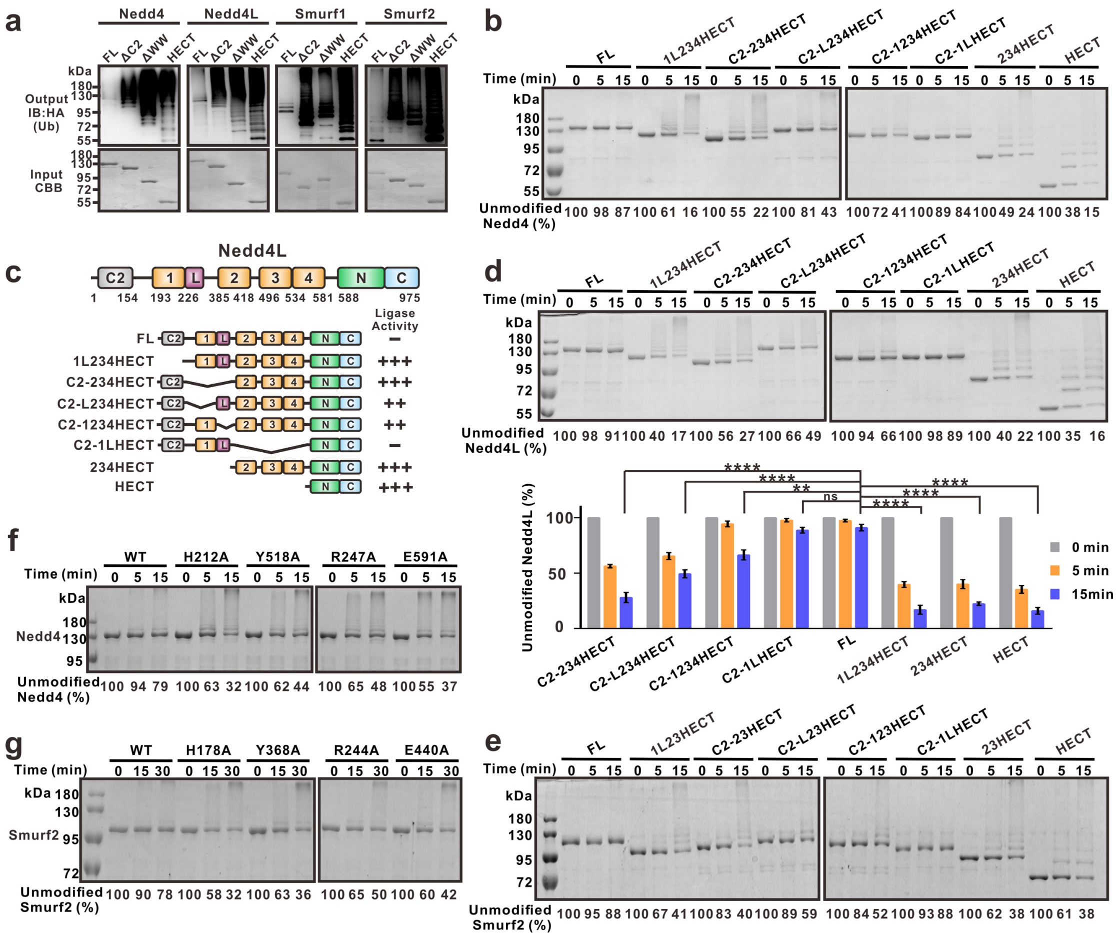

Supplementary Fig. 6 A varied dual-lock autoinhibition mechanism in Nedd4/4L and Smurf2. (**a**) In vitro ubiquitination assay of the C2 (ΔC2) or WW (ΔWW) region truncated mutants of Nedd4/4L and Smurf1/2. (**b**) Autoubiquitination assay of Trx-tagged full-length (FL) Nedd4 and various fragments. (**c**) Schematic of Nedd4L domains with summary enzymatic activity derived from autoubiquitination assay in (**d**). (**d**) Autoubiquitination assay of Trx-tagged full-length (FL) Nedd4L and various fragments. (**e**) Autoubiquitination assay of GST-tagged full-length (FL) Smurf2 and various fragments. (**f**) Autoubiquitination assay of Nedd4 mutants at the supposed WW1L-HECT interface. (**g**) Autoubiquitination assay of Smurf2 mutants at the supposed WW1L-HECT interface. Source data are provided as a Source Data file.

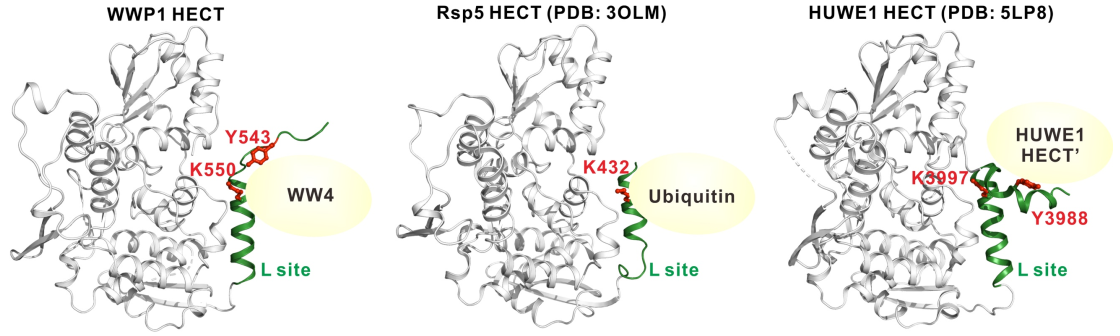

Supplementary Fig. 7 "Le" site may act as a versatile regulatory site on HECT domain. In this study, we showed that Tyr543^{WWP1} occupied the canonical PY motif binding site of WW4 and further locked WWP1 in its inactive state (left pannel). Ubiquitination of Rsp5 at Lys432 (corresponding to the conserved Lys550^{WWP1}) could suppress its activity by inducing its oligomerization. Similarly, the N-terminal extension of the HECT domain from non-Nedd4 E3 HUWE1 mediates its dimerization and thus keeps the E3 in low activity, and the conserved Tyr3988^{HUWE1} (corresponding to Tyr543^{WWP1}) provides a key contribution in dimer formation through hydrophobic interaction.

Supplementary Table 1. Constructs in this study.

Proteins	Constructs (aa)	Motif pattern	Referred to as
WWP1	1-922	C2-WW12L34-HECT	FL
WWP1	147-922	WW12L34-HECT	12L34HECT
WWP1	379-922	WW2L34-HECT	2L34HECT
WWP1	379-456+530-922	WW2L-HECT	2LHECT
WWP1	410-922	LWW34-HECT	L34HECT
WWP1	379-416+453-922	WW234-HECT	234HECT
WWP1	410-456+530-922	L-HECT	LHECT
WWP1	533-922	HECT	HECT
WWP1	1-147	C2	C2
WWP1	147-533	WW12L34	WW
WWP1	379-458	WW2L	WW2L
WWP1	346-383	WW1	WW1
WWP1	379-416	WW2	WW2
WWP1	410-458	L	L
WWP1	456-533	WW34	WW34
WWP1	410-533	LWW34	LWW34
WWP1	379-917	WW2L34-HECTΔCT	2L34HECTΔCT
WWP1	379-416+453-917	WW234-HECTΔCT	234HECTΔCT
WWP1	410-456+530-917	L-HECTΔCT	LHECTΔCT
WWP1	533-917	HECTΔCT	HECTΔCT
WWP2	1-870	C2-WW12L34-HECT	FL
WWP2	147-870	WW12L34-HECT	12L34HECT
WWP2	330-870	WW2L34-HECT	2L34HECT
WWP2	330-406+480-870	WW2L-HECT	2LHECT
WWP2	361-870	LWW34-HECT	L34HECT
WWP2	330-360+394-870	WW234-HECT	234HECT
WWP2	361-406+480-870	L-HECT	LHECT
WWP2	486-870	HECT	HECT
WWP2	147-480	WW12L34	WW
WWP2	331-404	WW2L	WW2L
WWP2	300-330	WW1	WW1
WWP2	331-361	WW2	WW2
WWP2	362-404	L	L
WWP2	405-475	WW34	WW34
WWP2	361-480	LWW34	LWW34
Itch	285-864	WW12L34-HECT	12L34HECT
Itch	285-400+470-864	WW12L-HECT	12LHECT
Itch	352-864	LWW34-HECT	L34HECT

Itch	481-864	HECT	HECT
Itch	143-474	WW12L34	WW
Nedd4	1-900	C2-WW1L234-HECT	FL
Nedd4	155-900	WW1L234-HECT	1L234HECT/△C2
Nedd4	1-182+340-900	C2-WW234-HECT	C2-234HECT
Nedd4	1-187+227-900	C2-LWW234-HECT	C2-L234HECT
Nedd4	1-223+348-900	C2-WW1234-HECT	C2-1234HECT
Nedd4	1-340+513-900	C2-WW1L-HECT	C2-1LHECT
Nedd4	345-900	WW234-HECT	234HECT
Nedd4	513-900	HECT	HECT
Nedd4	180-510	WW1L234	WW
Nedd4	1-182+510-900	C2-HECT	C2-HECT/△WW
Nedd4L	1-975	C2-WW1L234-HECT	FL
Nedd4L	164-975	WW1L234-HECT	1L234HECT/△C2
Nedd4L	1-179+365-975	C2-WW234-HECT	C2-234HECT
Nedd4L	1-189+230-975	C2-LWW234-HECT	C2-L234HECT
Nedd4L	1-225+386-975	C2-WW1234-HECT	C2-1234HECT
Nedd4L	1-377+588-975	C2-WW1L-HECT	C2-1LHECT
Nedd4L	385-975	WW234-HECT	234HECT
Nedd4L	588-975	HECT	HECT
Nedd4L	164-588	WW1L234	WW
Nedd4L	1-179+588-975	C2-HECT	C2-HECT/△WW
Smurf1	1-757	C2-WW1L2-HECT	FL
Smurf1	203-757	WW1L2-HECT	△C2
Smurf1	1-230+350-757	C2-HECT	△WW
Smurf1	366-757	HECT	HECT
Smurf2	1-748	C2-WW1L23-HECT	FL
Smurf2	145-748	WW1L23-HECT	1L23HECT/△C2
Smurf2	1-146+248-748	C2-WW23-HECT	C2-23HECT
Smurf2	1-146+193-748	C2-LWW23-HECT	C2-L23HECT
Smurf2	1-190+250-748	C2-WW123-HECT	C2-123HECT
Smurf2	1-250+354-748	C2-WW1L-HECT	C2-1LHECT
Smurf2	248-748	WW23-HECT	23HECT
Smurf2	365-748	HECT	HECT
Smurf2	145-371	WW1L23	WW
Smurf2	1-147+354-748	C2-HECT	C2-HECT/△WW

Supplementary Table 2. Primers used in this study.

Primer name	Primer sequences (5’-3’)
WWP1-1-Forward	CGGAATTCATGGCCACTGCTTCA
WWP1-147-Forward	GAGGAATTCGAAAATATAACAAACTGC
WWP1-346-Forward	GAC GAATTCGCCAACACAGAAAC
WWP1-379-Forward	CGGAATTCAGACCACAACCTTTA
WWP1-410-Forward	AGA GAATTCCAGCGGCCTACCATGG
WWP1-533-Forward	GAGGAATTCTCTGTAACTAAAGGT
WWP1-147-Reverse	TCTGTCGACTTATTCTTGCTCAATCAC
WWP1-383-Reverse	GAC GTCGACTCATAAAGGTTGTGGTC
WWP1-416-Reverse	GAG GTCGACTTA AGATTCCATGGTAGG
WWP1-533-Reverse	GAGGTCGACTTAAGATGACTTCCCATTG
WWP1-917-Reverse	TCCT GTCGACTTACTCTGTCTCT TCTATTGC
WWP1-922-Reverse	ACC GTCGACTTATTCTTGTCCAAATCC
WWP1-Δ417-452-Forward	CCTACCATGGAATCTGACCCTTATGGACC
WWP1-Δ417-452-Reverse	GGTCCATAAGGGTCAGATTCCATGGTAGG
WWP1-Δ457-529-Forward	AATGACCCTTATGGAGGGAAGTCATCTGTA
WWP1-Δ457-529-Reverse	TACAGATGACTTCCCTCCATAAGGGTCATT
WWP1-W409A-Forward	CCAGAACAACAACGGCACAGCGGCCTACCATG
WWP1-W409A-Reverse	CATGGTAGGCCGCTGTGCCGTTGTTGTTCTGG
WWP1-R427W-Forward	TGGCAATCTCAGTGGAACCAATTGCAG
WWP1-R427W-Reverse	CTGCAATTGGTTCCACTGAGATTGCCA
WWP1-F437A-Forward	GCTATGCAACAGGCTAACCAACGATAC
WWP1-F437A-Reverse	GTATCGTTGG TTAGCCTGTT GCATAGC
WWP1-Y441A-Forward	GTTTAACCAACGAGCCCTCTATTCGGC
WWP1-Y441A-Reverse	GCCGAATAGAGGGCTCGTTGGTTAAAC
WWP1-Y443A-Forward	CAACGATACCTCGCTTCGGCTTCAATG
WWP1-Y443A-Reverse	CATTGAAGCCGAAGCGAGGTATCGTTG
WWP1-S444L-Forward	CGATACCTCTAT CTGGCTTCAATGTTA
WWP1-S444L-Reverse	TAACATTGAAGCCAGATAGAGGTATCG
WWP1-M447A-Forward	CTATTCGGCTTCAGCGTTAGCTGCAGAA
WWP1-M447A-Reverse	TTCTGCAGCTAACGCTGAAGCCGAATAG
WWP1-E503A-Forward	CCAGAAGGCTGGGCAATTAGATATACT
WWP1-E503A-Reverse	AGTATATCTA ATTGCCCAGCCTTCTGG
WWP1-H517A-Forward	GTACTTTGTTGATGCTAACACAAGAACA
WWP1-H517A-Reverse	TGTTCTTGTGTTAGCATCAACAAAGTAC
WWP1-H517Y-Forward	GTACTTTGTTGATTAT AACACAAGAACA
WWP1-H517Y-Reverse	TGTTCTTGTGTTATAATCAACAAAGTAC

WWP1-Y543A-Forward	CCACAAATTGCTGCTGAACGCGGCTTT
WWP1-Y543A-Reverse	AAAGCCGCGTTCAGCAGCAATTTGTGG
WWP1-Y543E-Forward	GGTCCACAAATTGCTGAGGAACGCGGCTTTAGG
WWP1-Y543E-Reverse	CCTAAAGCCGCGTTCCTCAGCAATTTGTGGACC
WWP1-W549A-Forward	CGCGGCTTTAGG GCGAAGCTTGCTCAC
WWP1-W549A-Reverse	GTGAGCAAGCTTCGCCCTAA AGCCGCG
WWP1-F617E-Forward	CGAGAGAATGGTTTGAGTTGCTTTCACATGAAG
WWP1-F617E-Reverse	CTTCATGTGA AAGCAACTCA AACCATTCTCTCG
WWP1-M627E-Forward	GAAGTTTTGAACCCAGAGTATTGCTTATTTGAG
WWP1-M627E-Reverse	CTCAAATAAGCAATACTCTGGGTTCAAAACTTC
WWP1-P651A-Forward	TCAACCATTAATGCAGACCATCTTTCA
WWP1-P651A-Reverse	TGAAAGATGG TCTGCATTAATGGTTGA
WWP1-E697K-Forward	ATTAAGGATTTGAAATCTATTGATACT
WWP1-E697K-Reverse	AGTATCAATA GATTTCAAATCCTTAAT
WWP1-M804Q805A-Forward	GTTATGTTGTGTGGCGCAGCAGAGGTTGACTTGGC
WWP1-M804Q805A-Reverse	GCCAAGTCAACCTCTGCTGCGCCACACAACATAAC
WWP2-1-Forward	CGGAATTCATGGCATCTGCCAGC
WWP2-147-Forward	GAGGAATTCGATCTGGGAAATGTG
WWP2-285-Forward	GAGGAATTCAGCACTTCGGGTACA
WWP2-300-Forward	ATA GAATTCGACGCTCTGCCTGCTGG
WWP2-330-Forward	TAT GAATTCCGGCCCCTTCCTCCAGG
WWP2-331-Forward	ATA GAATTCCCCCTTCCTCCAGGCTGG
WWP2-361-Forward	GAGGAATTCCCGACCGCGGAGTAC
WWP2-362-Forward	ATA GAATTCACCGCGGAGTACGTGC
WWP2-405-Forward	ATA GAATTCGGCCCCCTCCCTCCT
WWP2-486-Forward	GAGGAATTCGGTTCCCCTGGTGC
WWP2-330-Reverse	ATA CTCGAGTTACCGCTCCCAGGTGGTGG
WWP2-361-Reverse	ATA CTCGAGTTACGGACGCTGCCAGGTGG
WWP2-404-Reverse	TAT CTCGAGTTACAGGGGATCATGGTCAGTCG
WWP2-475-Reverse	TCTCTCGAGTTAAGGATCCTTAAAGGT
WWP2-480-Reverse	TCTCTCGAGTTACTCAAACCCCGGGCG
WWP2-870-Reverse	CCCTCGAGTTACTCCTGTCC AAAGCC
WWP2-Δ361-393-Forward	ACCTGGCAGCGTTCTTCGAGTGCT
WWP2-Δ361-393-Reverse	AGCACTCGAAGAACGCTGCCAGGT
WWP2-Δ407-479-Forward	GATCCCCTGGGCCCCGAGTCGGGGACGAAG
WWP2-Δ407-479-Reverse	CTTCGTCCCCGACTCGGGGCCCAGGGGATC
WWP2-W358A-Forward	GACCACCACCGCGCAGCGTCCGA
WWP2-W358A-Reverse	TCGGACGCTGCGCGGTGGTGGTC
WWP2-H465A-Forward	CGATACTTTGTGGACGCCAATACCCGCACCACC
WWP2-H465A-Reverse	GGTGGTGCGGGTATTGGCGTCCACAAAGTATCG
WWP2-Y491A-Forward	GGTTCCCCTGGTGCT GCTGACCGCAGTTTTCGG

WWP2-Y491A-Reverse	CCGAAAACTGCGGTCAGCAGCACCAGGGGAACC
WWP2-M575E-Forward	GCTCAACCCTGAGTATTGTTTAT
WWP2-M575E-Reverse	ATAAACAATACTCAGGGTTGAGC
Itch-143-Forward	ATAGGATCCCAAGTAGAAGCTGAG
Itch-285-Forward	ATAGGATCCAGCCAAGCTCCCCTA
Itch-352-Forward	CGGGATCCTTGGAATCTGTCCGG
Itch-481-Forward	ATAGGATCCCCCCAGATAGCCTAT
Itch-474-Reverse	ATAGTCGACTTATTTTCCCGTTCGTGG
Itch-864-Reverse	ATAGTCGACTTACTCTTGTCCAAA
Itch-Δ401-469-Forward	GATCCGCTTGGTCCGCCACGAACGGGAAAATCA
Itch-Δ401-469-Reverse	TGATTTTCCCGTTCGTGGCGGACCAAGCGGATC
Itch-W347A-Forward	ACAAGAACCACAACAGCGCAGAGGCCAACCTTG
Itch-W347A-Reverse	CAAGGTTGGCCTCTGCGCTGTTGTGGTTCTTGT
Itch-H460A-Forward	CCATATTTTGTGGACGCCAATAGAAGAGCAACT
Itch-H460A-Reverse	AGTTGCTCTTCTATTGGCGTCCACAAAATATGG
Itch-Y485A-Forward	CCAGATAGCCGCTGTGCGGGACT
Itch-Y485A-Reverse	AGTCCCGCACAGCGGCTATCTGG
Itch-M569E-Forward	GAAGTGTTGAACCCAGAGTATTGCCTGTTTGAA
Itch-M569E-Reverse	TTCAAACAGGCAATACTCTGGGTTCAACACTTC
Nedd4-1-Forward	CGGAATTCATGGCAACTTGCGCGG
Nedd4-155-Forward	CGGAATTCAGTGGCTCAGAAGATG
Nedd4-180-Forward	CGGAATTCGCTGCTTGCCATTTGC
Nedd4-345-Forward	GAGGAATTCCCTACTTCATCTGGA
Nedd4-513-Forward	CGGAATTCGGACCAGCAGTGCCC
Nedd4-510-Reverse	CCCTCGAGTTATGCTACATTCTCCAAC
Nedd4-900-Reverse	CCCTCGAGTTAATCAACTCC ATCAAAGCCCTG
Nedd4-Δ183-339-Forward	ACCAGATGCTGCTTGCCTTC CTGTGCTTTTGC
Nedd4-Δ183-339-Reverse	GCAAAAGCACAGGAAGGCAAGCAGCATCTGGT
Nedd4-Δ188-226-Forward	CATTTGCAGCAACAAAACCTAACAGATGCT
Nedd4-Δ188-226-Reverse	AGCATCTGTTAGGTTTTGTTGCTGCAAATG
Nedd4-Δ224-347-Forward	TGGAAAAGACCAACCTCTGGATTACCACCA
Nedd4-Δ224-347-Reverse	TGGTGGTAATCCAGAGGTTGGTCTTTTCCA
Nedd4-Δ341-512-Forward	GAACAACCTACACTTGGACCAGCAGTGCCC
Nedd4-Δ341-512-Reverse	GGGCACTGCTGGTCCAAGTGTAGGTTGTTC
Nedd4-H212A-Forward	ACCTATTATGTAAACGCTGAATCTAGAAGAACA
Nedd4-H212A-Reverse	TGTTCTTCTAGATTCAGCGT TTACATAATAGGT
Nedd4-F244A-Forward	CGTGCAGCTACCACCAGGCGGCAGATATCCGAG
Nedd4-F244A-Reverse	GGTGGTAGCTGCACGTTGTGCTTGCAGTTGAAT
Nedd4-R247A-Forward	ACCACCGCTCGGCAGATATCCGAGGAAACAGAA
Nedd4-R247A-Reverse	CTGCCGAGCGGTGGTAAATGCACGTTGTGCTTG
Nedd4-H369A-Forward	TCATATTATG TAGATGCCAATTCCAGAACGACT

Nedd4-H369A-Reverse	AGTCGTTCTGGAATTGGCATCTACATAATATGA
Nedd4-H442A-Forward	CCTTTCTTTATTGACGCCAACACTAAAACCACC
Nedd4-H442A-Reverse	GGTGGTTTTAGTGTTGGCGTCAATAAAGAAAGG
Nedd4-H494A-Forward	ATCTTCTACATAAATGCCAATATAAAAAGAACA
Nedd4-H494A-Reverse	TGTTCTTTTTATATTGGCAT TTATGTAGAAGAT
Nedd4-E591A-Forward	GCCAGAGCTTGGTTCTTCCTGATCTCAAAGGAA
Nedd4-E591A-Reverse	GAACCAAGCTCTGGCAACTCCTCCATAATC CAA
Nedd4-F594E-Forward	GCCAGAGAATGGTTCGAACTGATCTCAAAGGAA
Nedd4-F594E-Reverse	TTCCTTTGAGATCAGTTCGAACCATTCTCTGGC
Nedd4-Y518A-Forward	GGACCAGCAGTGCCCGCCTCCAGGGATTACAA
Nedd4-Y518A-Reverse	TTGTAATCCCTGGAGGCGGGCACTGCTGGTCC
Nedd4L-1-Forward	GCGTCGACCCATGGCGACCGGGCTCGGGGAGCCGG
Nedd4L-164-Forward	GCGTCGACCCAGTGACCAGAGGGATG
Nedd4L-385-Forward	GCGTCGACCCCCGGGTCTGCCTTCA
Nedd4L-588-Forward	GCGTCGACCCGGTCCGGCTGTCCCTT
Nedd4L-588-Reverse	AAGCGGCCGCTTAACCAGTAATAGCTGG
Nedd4L-975-Reverse	TTGCGGCCGCTTAATCCACCCCTTCAAAT
Nedd4L-Δ180-364-Forward	GGAAGTTGTTGACTCATCATCAACTGTCACGG
Nedd4L-Δ180-364-Reverse	CCGTGACAGTTGATGATGAGTCAACAACTTCC
Nedd4L-Δ180-587-Forward	GTTGTTGACTCAGGTCCGGCTGTCCCT
Nedd4L-Δ180-587-Reverse	AGGGACAGCCGGACCTGAGTCAACAAC
Nedd4L-Δ190-229-Forward	CAGCACCAAGAGGAATCCTCGGAGTCGGAC
Nedd4L-Δ190-229-Reverse	GTCCGACTCCGAGGATTCCTCTTGGTGCTG
Nedd4L-Δ226-385-Forward	TGGCACAGACCAAGCGGTCTGCCTTCAGGC
Nedd4L-Δ226-385-Reverse	GCCTGAAGGCAGACCGCTTGGTCTGTGCCA
Nedd4L-Δ378-587-Forward	GAACCAACGCCATCAGGTCCGGCTGTCCCT
Nedd4L-Δ378-587-Reverse	AGGGACAGCCGGACCTGATGGCGTTGGTTC
Smurf1-1-Forward	CCAAGCTTATGTCGAACCCCGGG
Smurf1-203-Forward	CCAAGCTTCAAGATCAAAGACTTC
Smurf1-366-Forward	CGGAATTCGACGAGGAGCTTCCTG
Smurf1-757-Reverse	CCCTCGAGTTACTCCACAGCAAACCC
Smurf1-Δ231-349-Forward	AACCGACCACACGGCAAGGAGCCCAGCCAG
Smurf1-Δ231-349-Reverse	CTGGCTGGGCTCCTTGCCGTGTGGTCGGTT
Smurf2-1-Forward	CGGAATTCATGTCTAACCCCGGA
Smurf2-145-Forward	CGGAATTCGGAGGACAAGTTGTG
Smurf2-248-Forward	CGGAATTCCATACTCCTCCAGAC
Smurf2-365-Forward	CGGAATTCGTCCCAAGGTACAAG
Smurf2-371-Reverse	GCGTCGACTTAGTCTCGCTTGTACCT
Smurf2-748-Reverse	GCGTCGACTTATTCCACAGCAAATCC
Smurf2-Δ147-192-Forward	ATAGGCACAGGAGGATCCGAATATTCTAGC
Smurf2-Δ147-192-Reverse	GCTAGAATAT TCGGATCCTCCTGTGCCTAT

Smurf2-Δ147-247-Forward	ATAGGCACAGGAGGACATACTCCTCCAGAC
Smurf2-Δ147-247-Reverse	GTCTGGAGGAGTATGTCCTCCTGTGCCTAT
Smurf2-Δ148-353-Forward	GGCACAGGAGGACAATCGTTATGTCCTGATG
Smurf2-Δ148-353-Reverse	CATCAGGACATAACGATTGTCCTCCTGTGCC
Smurf2-Δ191-249-Forward	GAGCGCCCAACACGACCTCCAGACCTACCA
Smurf2-Δ191-249-Reverse	TGGTAGGTCTGGAGGTCGTGTTGGGCGCTC
Smurf2-Δ251-353-Forward	CATTTACATACTCCTTCGTTATGTCCTGAT
Smurf2-Δ251-353-Reverse	ATCAGGACATAACGAAGGAGTATGTAAATG
Smurf2-H178A-Forward	ATCCAGTATCTAAACGCTATAACAAGAACTACG
Smurf2-H178A-Reverse	CGTAGTTCTTGTTATAGCGTTTAGATACTGGAT
Smurf2-Y241A-Forward	AGAAATGCTATGAGCAGAACACATTTACATACT
Smurf2-Y241A-Reverse	GCTCATAGCATTTCTATGTCGTTGTGACCTGAC
Smurf2-R244A-Forward	ATGAGCGCTACACATTTACATACTCCTCCAGAC
Smurf2-R244A-Reverse	ATGTGTAGCGCTCATGTAATTTCTATGTCGTTG
Smurf2-H271A-Forward	CAGGTGTATTTCTTAGCTACACAGACTGGTGTG
Smurf2-H271A-Reverse	CACACCAGTCTGTGTAGCTAAGAAATACACCTG
Smurf2-H318A-Forward	GTTTATTTCGTTGACGCTAACAACAGAACAACA
Smurf2-H318A-Reverse	TGTTGTTCTGTTGTTAGCGTCAACGAAATA AAC
Smurf2-Y368A-Forward	CTGACAGTCCCAAGGGCCAAGCGAGACCTGGTTC
Smurf2-Y368A-Reverse	GAACCAGGTCTCGCTTGGCCCTTGGGACTGTCAG
Smurf2-E440A-Forward	GCCAGGGCTTGGTTGTATCTCTTGTCACATGAA
Smurf2-E440A-Reverse	CAACCAAGCCCTGGCAACGCCTCCATAGTCAAG
Smurf2-Y443E-Forward	GCCAGGGAATGGTTGGAGCTCTTGTCACATGAA
Smurf2-Y443E-Reverse	TTCATGTGACAAGAGCTCCAACCATTCCCT GGC
