# Supplementary material for: A multi-lock inhibitory mechanism for fine-tuning enzyme activities of the HECT family E3 ligases
Source: Nat Commun. 2019 Jul 18;10:3162. doi: 10.1038/s41467-019-11224-7 (PMC6639328; doi:10.1038/s41467-019-11224-7)
Supplement: Supplementary file 3 — Reporting Summary [file 41467_2019_11224_MOESM3_ESM.pdf]

nature research

Corresponding author(s): Wenyu Wen

Last updated by author(s): Jun 27, 2019

Reporting Summary

Nature Research wishes to improve the reproducibility of the work that we publish. This form provides structure for consistency and transparency in reporting. For further information on Nature Research policies, see Authors & Referees and the Editorial Policy Checklist.

Statistics

For all statistical analyses, confirm that the following items are present in the figure legend, table legend, main text, or Methods section.

n/a	Confirmed	
☐	☒	The exact sample size (*n*) for each experimental group/condition, given as a discrete number and unit of measurement
☐	☒	A statement on whether measurements were taken from distinct samples or whether the same sample was measured repeatedly
☐	☒	The statistical test(s) used AND whether they are one- or two-sided<br>*Only common tests should be described solely by name; describe more complex techniques in the Methods section.*
☒	☐	A description of all covariates tested
☒	☐	A description of any assumptions or corrections, such as tests of normality and adjustment for multiple comparisons
☐	☒	A full description of the statistical parameters including central tendency (e.g. means) or other basic estimates (e.g. regression coefficient) AND variation (e.g. standard deviation) or associated estimates of uncertainty (e.g. confidence intervals)
☐	☒	For null hypothesis testing, the test statistic (e.g. *F*, *t*, *r*) with confidence intervals, effect sizes, degrees of freedom and *P* value noted<br>*Give P values as exact values whenever suitable.*
☒	☐	For Bayesian analysis, information on the choice of priors and Markov chain Monte Carlo settings
☒	☐	For hierarchical and complex designs, identification of the appropriate level for tests and full reporting of outcomes
☒	☐	Estimates of effect sizes (e.g. Cohen's *d*, Pearson's *r*), indicating how they were calculated

Our web collection on statistics for biologists contains articles on many of the points above.

Software and code

Policy information about availability of computer code

Data collection	N/A
Data analysis	Data analysis was carried out in HKL2000(HKL Research Inc.), Prism 6.02 (GraphPad)

For manuscripts utilizing custom algorithms or software that are central to the research but not yet described in published literature, software must be made available to editors/reviewers. We strongly encourage code deposition in a community repository (e.g. GitHub). See the Nature Research guidelines for submitting code & software for further information.

Data

Policy information about availability of data

All manuscripts must include a data availability statement. This statement should provide the following information, where applicable:

- Accession codes, unique identifiers, or web links for publicly available datasets
- A list of figures that have associated raw data
- A description of any restrictions on data availability

All the relevant data supporting the findings of this study are available within the article, or from the corresponding author on reasonable request. The structure data for WWP1 2L34HECT and L34HECT have been deposited in the Protein Data Bank with PDB ID code 6J1X and 6J1Y, respectively.

Field-specific reporting

Please select the one below that is the best fit for your research. If you are not sure, read the appropriate sections before making your selection.

☒ Life sciences ☐ Behavioural & social sciences ☐ Ecological, evolutionary & environmental sciences

Life sciences study design

All studies must disclose on these points even when the disclosure is negative.

Sample size	All experiments were repeated three times.
Data exclusions	No data was excluded.
Replication	All assays were repeated three times showing similar results.
Randomization	All experiments were repeated three times.
Blinding	We were blinded to group allocation during data collection and analysis.

Reporting for specific materials, systems and methods

We require information from authors about some types of materials, experimental systems and methods used in many studies. Here, indicate whether each material, system or method listed is relevant to your study. If you are not sure if a list item applies to your research, read the appropriate section before selecting a response.

Materials & experimental systems

n/a	Involved in the study	
☐	☒	Antibodies
☐	☒	Eukaryotic cell lines
☒	☐	Palaeontology
☒	☐	Animals and other organisms
☒	☐	Human research participants
☒	☐	Clinical data

Methods

n/a	Involved in the study	
☒	☐	ChIP-seq
☒	☐	Flow cytometry
☒	☐	MRI-based neuroimaging

Antibodies

Antibodies used	anti-Flag (ABclonal, AE005), anti-p63 (CST, 13109S), goat anti-mouse (ABclonal, AS003), goat anti-rabbit antibody (ABclonal, AS014).
Validation	All the antibodies used in the present study are well verified commercial antibodies

Eukaryotic cell lines

Policy information about cell lines

Cell line source(s)	HEK293T cells and MCF-10A cells (from ATCC) were used in this study
Authentication	All the cell lines are commercial and authenticated
Mycoplasma contamination	No mycoplasma contamination was detected in HEK293T and MCF-10A cells used in this study.
Commonly misidentified lines (See ICLAC register)	No commonly misidentified cell lines were used.
